# Organoids with a Type 1 Collagen Scaffold to Model Bacterial Cancer Therapy

**DOI:** 10.3390/cells14070524

**Published:** 2025-04-01

**Authors:** Lydia Farrell, Cleo Bonnet, Alethea Tang, Severina Peneva, Non G. Williams, Sunil Dolwani, Lee Parry, Paul Dyson

**Affiliations:** 1 Institute of Life Science, Swansea University Medical School, Singleton Park, Swansea SA2 8PP, UK; l.e.l.farrell@swansea.ac.uk (L.F.); cleo.bonnet@bd.com (C.B.); a.m.tang@swansea.ac.uk (A.T.); 901826@swansea.ac.uk (S.P.); 2 European Cancer Stem Cell Research Institute, School of Biosciences, Hadyn Ellis Building, Cardiff University, Maindy Road, Cardiff CF24 4HQ, UK; williamsng1@cardiff.ac.uk; 3 School of Medicine, Cardiff University, Cardiff and Vale University Health Board, Cardiff CF14 4XN, UK; dolwanis@cardiff.ac.uk

**Keywords:** bacterial cancer therapy, organoid, *Salmonella enterica* serovar Typhimurium, personalised medicine, type 1 collagen

## Abstract

Bacterial cancer therapy (BCT) is emerging as an important option for the treatment of solid tumours, with promising outcomes in preclinical trials. Further progress is hampered by an incomplete understanding of how oncotropic bacteria, such as attenuated strains of *Salmonella enterica* serovar Typhimurium, colonise tumours and the responses of both the bacteria and tumour cells to this colonisation. To model this, we developed organoids that are permissive for bacterial colonisation, replacing the conventional commercially available extracellular matrix (e.g., Matrigel) with a type I collagen matrix scaffold. A comparison of the two extracellular matrices indicated that type 1 collagen permitted an initial infection efficiency more than 5-times greater than with Matrigel. In addition, subsequent growth within type 1 collagen expanded bacterial cell numbers by over 10-fold within 4 days of infection. These organoids allow for the visualisation of bacterial chemoattraction, cell invasion and subsequent population of the interior lumen, and will permit the future optimisation of BCT. In addition, by establishing patient-derived organoids, we demonstrate a platform for developing future personalised treatments exploiting BCT.

## 1. Introduction

Immortalised cell lines provide cheap, easily expandable and reproducible systems that can be grown in vitro [1]. Traditionally, these cells have been grown on a flat plane as 2D cell cultures. However, growing on a flat surface does not represent how cells grow and function in the human body, where they are surrounded by other cells in three dimensions, and 2D models fail to mimic important epithelial functions [2]. In contrast, 3D cell culture systems are more complex and better mimic the architecture of the in vivo environment. The identification of progenitor stem cells and their ability to create 3D long-term differentiated organoids [3] have provided a platform with the structural properties of tissue and interactions with an extra cellular matrix, both with cell lines and expandable primary cells.

The growth of organoids is dependent on a 3D scaffold, typically provided by an extracellular matrix (ECM), in which cells can survive, grow and proliferate. ECMs also provide essential cues to cells, making them crucial for the establishment of physiologically relevant 3D tissue cultures. Commonly used ECMs such as Matrigel are complex, containing over 1800 distinct proteins, and this complexity together with batch variations contributes to a poor understanding of organoid development [4]. One property of many commercial mammalian-derived ECMs is the inclusion of the antibiotic gentamicin (50 mg/mL in Matrigel) to inhibit bacterial contamination, although the presence of the antibiotic is not explicitly stated in the product description.

A corollary is that the presence of the antibiotic can prevent the use of these ECMs for studying how cell-invasive bacteria can infect organoid cells. Intestinally derived organoids typically develop an apical and basolateral luminal side. To avoid the problem with gentamicin and model intestinal luminal infection, studies have employed the microinjection of bacteria into the luminal space [5,6,7,8]. This approach avoids the need for the bacteria to persist and invade through the gentamicin-containing Matrigel. For example, the potential of organoids as a model for studying host–microbial interactions has been demonstrated using human gastric organoids and the pathogenic microbe *Helicobacter pylori* [9,10]. This bacterium is known to cause infection, ulceration, and gastric cancer in humans [11]. Apical infection of the gastric organoids with *H. pylori* recapitulates the known hallmarks of infection, including a marked increase in mucus-secreting cell expression, induction of cytokine production via the up-regulation of NF-κB (known to induce gastritis), and production of the *H. pylori* virulence factor CagA (cytotoxin-associated gene A). The latter is associated with mutagenesis and tumour progression. Thus, these studies underline how organoids can offer a valuable near-physiological modelling system in which to begin deciphering the complex interactions between the host epithelium and infectious agents.

Another study has reported an observable bacterial infection of ‘inside-out’ intestinal organoids prepared with a Cultrex basement membrane extract containing gentamicin as an ECM; by reversing apical polarity, an antibiotic-free epithelial layer was presented on the exterior [12]. However, we were not able to observe the efficient infection of typical normal-polarity Matrigel-grown organoids with *Salmonella enterica* strain SL7207, an attenuated tumour-targeting strain that has been employed for bacterial cancer therapy (BCT) [13,14]. This is despite having observed both good invasion of cell lines cultured in 2D and the colonisation of tumours in mice after intraperitoneal injection of the bacteria, remote from the site of the tumours [14]. Here, we describe using gentamicin-free type 1 collagen as an ECM for both immortalised cell-derived organoids and primary patient organoids. These organoids can permit studies into chemotaxis and cell invasion by oncotropic bacteria such as SL7207. Moreover, patient-derived organoids can permit future testing of the efficacy of any therapeutic payload that can be delivered by the bacteria.

## 2. Materials and Methods

### 2.1. Bacterial Strains and Plasmids

*E. coli* strain JM109 [15] was used as a host for development of derivatives of plasmid RSF1010. *Salmonella enterica* serovar Typhimurium strain SL7207 [16] was used as the oncotropic bacterium in this study. Bacterial cultures were grown from glycerol stocks in a shaking incubator at 37 °C, 200 rpm overnight in 5 mL of autoclaved Luria-Bertani (LB) broth (25 g LB broth powder (Melford, Ipswich, UK) in 1 L deionised H_2_O (dH_2_O)) containing 5 µL of 50 μg/mL Apramycin (Apr50) when needed for selection of plasmid-containing strains. For co-culture with human cells, cultures were then expanded and grown under the same conditions in 250 mL of LB broth containing 250 μL of Apr50 and inoculated with 2.5 mL of bacterial culture (1/100 volume) until an appropriate OD_600_ was reached. Plasmids used in this study are listed in Table 1.

### 2.2. Generation of a Stable RSF1010-Derived Plasmid for GFP Expression

RSF1010 is a naturally occurring plasmid of *Salmonella enterica*. Its presence in SL7207 strains in the absence of selection and after many manipulations indicates it is very stable. To exploit RSF1010 as a stable expression vector, an apramycin (Apr) resistance gene was amplified from plasmid pIJ8600 [19], using primers Apr(EcoRI) forward and reverse (Table 2), and cloned at the unique *Eco*RI site of RSF1010. The size of the resulting plasmid was reduced by deleting a non-essential *Pst*I fragment to the left and non-essential *Nru*I-*Pvu*II fragments to the right of the Apr resistance gene, creating pLF300. The copy number of the resulting plasmid was low and plasmid yields poor as much of the DNA was nicked at the plasmid *oriT* sequence by the Mob proteins [20]. Site-directed mutagenesis, using a Q5 site-directed mutagenesis kit (New England Biolabs, Ipswich, MA, USA) and forward and reverse primers 0223201 to introduce a 4 bp deletion in *mobC* (mobility protein and repressor of promoters P1 and P3), increased both the copy number to ~50 and yields of supercoiled pLF301, due to increased expression of *rep* genes (Supplementary Figure S1B). Incorporation of a GFP gene, amplified from plasmid pdagGFP [18], at the unique *Pst*I site of pLF301, generating plasmid pEG200, enabled visualisation of plasmid-containing bacteria in a co-culture setting (Supplementary Figure S1). The new plasmid was unable to displace resident RS1010 in SL7207; i.e., no Apr^R^ clones were obtained after electroporation with plasmid pEG200 isolated from *E. coli*. Displacement of RSF1010 was achieved by first constructing a derivative of pUC18 containing the apramycin resistance gene inserted at the unique *Bam*HI site (after amplification from pIJ8600, using primers Apr (BamHI) forward and reverse). A pUC18-Apr::RSF1010 fusion plasmid was then constructed by inserting pUC18-Apr linearized with *Eco*RI at the unique *Eco*RI site of RSF1010. This fusion plasmid was electroporated into SL7207, resulting in the successful displacement of RSF1010 by virtue of double antibiotic selection (ampicillin and apramycin) for the fusion plasmid and its high copy number. The stability of the fusion plasmid after 10 generations of non-selective growth was 2.3 × 10^−4^, which permitted the isolation of plasmid-free SL7207. This plasmid-free strain was subsequently electroporated to introduce pEG200.

### 2.3. Cell Lines

Two-dimensional co-culture assays used HT29 (ATCC HTB-38), Caco2 (ATCC HTB-37), SW480 (ATCC CCL-228) epithelial colorectal adenocarcinoma, and HCT116 (ATCC CCL-247) epithelial colorectal carcinoma cell lines. Three-dimensional cell culture assays used colorectal cancer organoid ISO50 cells (Cat. No. OES-ISO50-CXP1; Molecular Devices, Wokingham, UK).

### 2.4. Primary Tissues

Primary patient samples were collected from the approved SERRATED POLYP PROGNOSTIC MARKERS STUDY—SAPPER under IRAS approval 241951. The trial protocol was approved by the Health Research Authority (HRA) and a Multi-Centre Research Ethics Committee (REC reference 20/WA/0021) and was sponsored by Cardiff University.

### 2.5. 2D Cell Line Co-Culture

Strains of SL7207 were grown at 37 °C in LB with relevant antibiotic selection. OD600 readings were taken and used to calculate viable bacterial number. Colon cancer cells were seeded at 1.6 × 10^5^ per well in ibidi 24 well plates (IB-82426) and incubated for 48 h at 37 °C for cells to adhere and recover. All cells were cultured in Dulbecco’s Modified Eagle Medium (DMEM; Gibco 41965-039, Waltham, MA, USA), supplemented with 10% fetal bovine serum (FBS; Gibco 17479633) and 1% PenStrep (Gibco 15140122). To determine cell numbers, cancer cells were detached using trypsin (Gibco 25200072) digestion and resuspended in a known volume of cell culture media. Using 10 µL trypan blue (Sigma, St. Louis, MO, USA) to 10 µL cell suspension, cells were counted on a 100 µm haemocytometer, and the number of cells per ml was quantified. Using both cell counts, the resuspension dilution and volume required for a multiplicity of infection (MOI) of 1:1000 cancer cells to bacteria were obtained. Bacteria were centrifuged at 1600× *g* at 4 °C for 10 min, and LB was removed and washed with 1 mL PBS. Centrifugation was repeated, PBS removed, and bacteria resuspended in the required volume of DMEM, 10% FBS, Apr50 to achieve the MOI of 1:1000 cancer cells to bacteria.

Cell culture media were removed from cancer cells and replaced with 500 µL SL7207 DMEM Apr50. Co-cultures were incubated at 37 °C, 5% CO_2_ for 2 h. After 2 h, extracellular media were removed, and cells were gently washed with prewarmed (37 °C) PBS that was subsequently replaced with DMEM containing 200 μg/mL gentamicin to kill any extracellular SL7207. The cells were then incubated for 1 h at 37 °C, 5% CO_2_, prior to fixation and or staining for immunofluorescence confocal microscopy.

### 2.6. 3D Cell Culture: Seeding of ISO50 Organoids

Cell lines were cultured in ISO50 media (Table S1); briefly, 7.5 mL each of 1 M HEPES (Gibco 15630056) to give a final concentration of 15 mM, 200 mM Glutamax (Gibco 35050038) and 10,000 units/mL penicillin-streptomycin (Gibco 15140122) were added to 500 mL of Advanced DMEM/F12 (Gibco 126340228). For complete medium, this was supplemented with 5 mL of 100× N2 supplement (Gibco 17502048), 10 mL of 50× B27 supplement (Gibco 17504044), and 500 µL of 1 M N-acetyl cysteine, mixed well, and stored in 25 mL aliquots at −20 °C. Once thawed, the complete media were stored at 4 °C for up to two weeks.

PBS and ISO50 medium were warmed to 37 °C, a Matrigel aliquot was retrieved from the −20 °C freezer and placed on ice, and a 24-well plate was placed in the incubator to warm to 37 °C. ISO50 or Caco2 cells were retrieved from liquid nitrogen and thawed in a water bath for 1 min, transferred to a 15 mL Falcon tube, and 4 mL of warm PBS was added. This was centrifuged at 300× *g* for 5 min, the supernatant poured off, followed by a quick spin to collect any residual liquid. After removal of any remaining supernatant with a pipette, the pellet was resuspended in 400 µL of ISO50 media for a 1:8 split (volume adjusted for other split ratios, e.g., 200 µL for 1:4). The cells were gently pipetted to avoid bubbles, and the desired cell volume was mixed with Matrigel in a 50:50 ratio (e.g., 175 µL cells + 175 µL Matrigel for 6 domes). Using a 24-well plate, 50 µL domes were dispensed into each well, allowing them to set for a few minutes, and then the plate was transferred to a 37 °C 5% CO_2_ incubator for 20 min before adding 500 µL per well of ISO50 complete media with 10 µM ROCK inhibitor (StemCell #72304), changing the media every two days. Organoids were cultured for one week.

### 2.7. Passaging of Organoids

After a week for organoid establishment, examination under a microscope should ideally reveal large organoids with few dark lumens. If the lumens were dark, a harsh split was performed, such as 1:20; otherwise, a 1:16 split was sufficient. Consequently, to seed up to 16 domes, 1 original dome was dissociated. The media were removed from the well; then, 500 µL of cold PBS was added and pipetted up and down to fully dissociate the dome, and then the suspension was transferred to a 15 mL Falcon tube. This was repeated with an additional 500 µL of PBS, then topped up to 5 mL with PBS, and centrifuged at 300× *g* for 5 min. After spinning, the PBS was discarded, leaving approximately 1 mL at the bottom to resuspend the pellet by pipetting. Then, fresh PBS was added up to 5 mL and spin repeated. The remaining supernatant was removed and 400 µL of ISO50 medium was then added with gentle pipetting to break up any cell clumps without creating bubbles. The volume was adjusted to permit plating 20,000 cells per well. For a 50:50 cell-to-Matrigel ratio, 75 µL of cell suspension was mixed with 75 µL of Matrigel. In a labelled 24-well plate, 50 µL of the cell–Matrigel mix was added to the centre of each well, briefly allowed to set, with the plate transferred to the incubator for 20 min, allowing the domes to set, before adding 500 µL per well of media with 10 µM ROCK inhibitor. Organoids were cultured for one week before infection. Jellagel or collagen was prepared as described below and used in the place of Matrigel at the passaging phase.

### 2.8. Jellagel Domes

To prepare Jellagel domes, a Jellagel (Jellagen JGEL10 ML, a collagen derived from the jellyfish *Rhizostoma pulmo*) solution was gently mixed with its buffer, using 120 µL of buffer for every 1 mL of Jellagel, an hour before assembling. Thorough mixing was achieved by inverting the tube, avoiding bubbles, and avoiding vortexing. When dilution was required, 0.5 mL of PBS per 1 mL of Jellagel was added for up to a 1/3 dilution. Subsequently, the mixture was incubated at room temperature for an hour, adding 100 µL of thawed crosslinker solution per 1 mL of Jellagel (before any dilution) and mixing thoroughly without vortexing or creating bubbles. The mixture was rested for 2–3 min; then, a prepared cell pellet was carefully resuspended in the Jellagel solution. Immediately, 50 µL of the cell/Jellagel mix was dispensed into each well of a 24-well plate and allowed to set at room temperature for 15 min. The domes were then incubated at 37 °C for 30 min to complete hydrogel formation. Finally, the domes were covered with 0.5 mL of media and the plate returned to the 37 °C incubator.

### 2.9. Collagen Preparation

The procedures we used for generating collagen organoids were based on previously published methods [21,22]. Type 1 collagen powder (Sigma C7661) was prepared fresh to achieve a final concentration of 2 mg/mL collagen. Thus, 10 mg of collagen was dissolved in 5 mL of 7 mM glacial acetic acid (GAA) (4 µL of GAA to 10 mL of distilled water (dH_2_O), filter sterilized), in a 15 mL Falcon tube, sealed with Parafilm and left on a roller at 4 °C overnight, avoiding shaking. Once fully dissolved, the collagen solution was stored at 4 °C for future use.

To prepare neutralization buffer for diluting 2 mg/mL type 1 collagen, we used a buffer containing HEPES (40 mM), sodium bicarbonate (NaHCO_3_, 106 mM), sodium hydroxide (NaOH, 15.3 mM), and PBS [19], using the following stock solutions: 1 M NaHCO_3_ (8.4 g in 100 mL dH_2_O) and 1 M NaOH (4 g in 100 mL dH_2_O), to 83.87 mL of 1× PBS, add 4 mL of 1 M HEPES, 10.6 mL of 1 M NaHCO_3_, and 1.53 mL of 1 M NaOH. The solutions were mixed well, filter sterilised and stored at 4 °C.

For use, we diluted 2 mg/mL type I collagen to 1 mg/mL by mixing 500 µL of 2 mg/mL collagen with 500 µL of neutralization buffer. Then, 800 µL of this mixture was used to resuspend an organoid pellet for a 1:16 split. Using a cold pipette tip, 50 µL of organoid–collagen domes was dispensed into the centre of wells in a pre-warmed 24-well plate. The domes were allowed to set slightly for a few minutes, and then the plate was carefully transferred to a 37 °C incubator and left for 30 min. Afterwards, 500 µL of ISO50 or media was added to each dome. The organoids were incubated and fed for six days.

### 2.10. Isolation of Crypts from Patient Samples

Biopsy samples were collected in 10 mL of cold phosphate-buffered saline (PBS) containing 4× penicillin-streptomycin and 1 µg/mL Fungizone, washed in 10 mL of the same solution, then twice more in PBS with 1× penicillin-streptomycin and 1 µg/mL Fungizone. After allowing the tissue to settle due to gravity, it was gently broken up using a 1 mL filter tip. The mixture was briefly centrifuged at 290× *g* for 1 min at 4 °C, and the supernatant was aspirated off, prior to adding 5 mL of Gentle Cell Dissociation Reagent (StemCell #07174). The mixture was incubated on a roller in a cold room for 30 min, centrifuged again, and the supernatant was removed via aspiration. To prevent crypts from sticking to pipette tips, the tips were pre-wetted with DMEM/F12 containing 1% BSA; then, the tissue was resuspended in 1 mL of this solution. Vigorous pipetting up and down 20 times released crypts, and the suspension was passed through a 70 µm strainer into a new tube, rinsing with an additional 1 mL of DMEM/F12 + 1% BSA.

For organoid cultures, the crypt count was first determined by placing three 10 µL aliquots on a slide, counting under a microscope, and, from that, calculating the total number. This helped to determine the number of culture domes needed, with each dome containing 1000 crypts. The sample was centrifuged at 200× *g* for 5 min, leaving 100 µL of supernatant, and then Matrigel in DMEM/F12 + 1% BSA was added to achieve a 1:1 mixture. Further, 50 µL of this crypt suspension per dome was dispensed into a pre-warmed 24-well plate. The plate was incubated at 37 °C for 20 min to solidify. IntestiCult™ Organoid Growth Medium (Stemcell #6010) with ROCK inhibitor for primary culture was prepared, and 500 µL of this medium was added to each dome. Every two days, the medium was refreshed with IntestiCult™ without ROCK inhibitor. Plates were incubated at 37 °C with 5% CO_2_ to support organoid growth.

To establish tumour lines, we used ISO50 media as an expansion medium depleted of Wnt3A-CM or Wnt surrogate. In this way, tumour cells with acquired Wnt pathway independence could be selected in culture.

### 2.11. Co-Culture of 3D Organoids

For bacterial co-culture, crypt organoids were first established in Matrigel and then passaged into type 1 collagen, as described above. Collagen organoid domes were grown for 6 days prior to addition of bacteria. To establish cell number, a representative organoid was selected, gently washed, and 20 µL of 2.5 mg Liberase (Roche, Basel, Switzerland) in 480 µL ISO50 media was added and incubated at 37 °C for 20 min. After 20 min, the dome was broken down by pipetting up and down and transferred to a 15 mL centrifuge tube. The well was rinsed with 500 µL PBS, which was also transferred to the centrifuge tube along with a further 4 mL of PBS. This was centrifuged at 300× *g* for 5 min, the supernatant removed, and 1 mL TrypLE (Gibco 12604) added to the pellet. After 15 min incubation at 37 °C, 9 mL of ISO50 media was added, and the cell suspension was strained through a 40 µm cell strainer. The flow through was diluted with a further 5 mL ISO50 media and centrifuged at 300× *g* for 5 min. After centrifugation, all media were carefully removed and the pellet resuspended in 1 mL fresh media. This was used to count cells with Trypan blue, as described above. These data were used to calculate the required bacterial cell number for an MOI of 1:1000.

### 2.12. Immunofluorescence Imaging

Imaging of cells grown in 2D cultures was performed essentially using steps described previously [20]. To perform immunofluorescence (IF) on organoids, the media were carefully removed and 500 µL of 2% paraformaldehyde (PFA) added to fix the samples for 30 min. The PFA solution was removed and the samples washed twice with 500 µL of PBS, allowing each wash to sit for 5 min. The samples were permeabilized by adding 500 µL of 0.1% Triton/PBS for 10 min, then washed twice more with PBS, each for 5 min. Blocking was achieved by adding 250 µL of a 10% goat serum blocking buffer for one hour; this buffer was prepared by mixing 100 µL of goat serum with 900 µL of PBS containing 0.1% Tween.

To perform a Phalloidin 488 actin stain on cells, the Phalloidin 488 stain was diluted 1:20 in PBS. This was added to the cells and incubated for 30 min to stain the actin filaments. Subsequently, the cells were washed three times with PBS for 5 min per wash, then mounted with DAPI (Vectashield DAPI, Vector Laboratories, Newark, NJ, USA) for nuclear visualization. Immunofluorescence images were acquired on a Zeiss LSM 880 and an LSM 980 confocal microscope and processed using ZEN lite software (ZEN 3.4 blue edition) to obtain mean fluorescence intensity (MFI) per field.

### 2.13. Statistical Analyses

Using Zeiss Zen software, fluorescence intensities were extracted per field. A lower threshold fluorescence intensity, MFI and an average bacterial size were used to convert these values to use fluorescence intensity as a proxy for bacterial number. This was validated against multiple manual counts. Statistical analyses were performed using GraphPad Prism Version 10.4. Comparisons between two conditions used unpaired t-test, and, for rank testing of multiple groups, a Kruskal–Wallis test was employed, with a threshold for significance set at *p* < 0.05 in both.

## 3. Results

### 3.1. Optimisation of Organoid Infection

**Figure 1:**
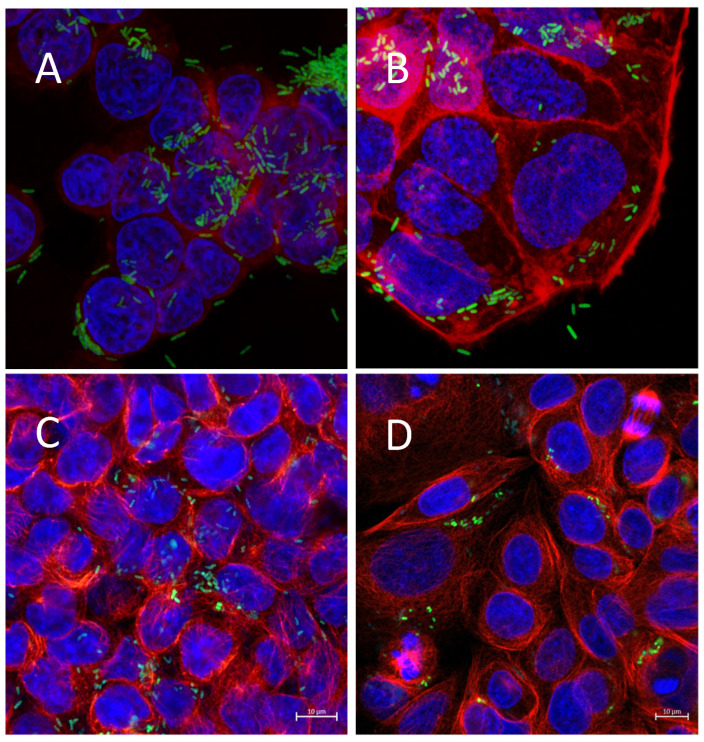
Infection of 2D colon cell line cultures with SL7207 GFP bacteria (**A**) HT29 cells, (**B**) Caco2 cells, (**C**) HCT116 cells, (**D**) SW480 cells. Cells were infected with SL7207-GFP for 1 h followed by gentamycin treatment. Cells are stained with DAPI (blue), actin (red), and SL7207-GFP (green).

In a 2D cell culture set up, good invasion by SL7207 of colon cancer cell lines was observed (Figure 1). A novel plasmid system using the conserved endogenous *Salmonella* plasmid RSF1010 as a platform for a high-copy-number cloning vector enabled stable tagging of the SL7207 strain with high expression of the GFP protein (Figure S1). Employing confocal microscopy with fluorescent staining of actin/tubulin and DAPI to mark the cell structure, the GFP bacteria can be seen to surround and invade all four cancer cell lines used (Figure 1).

**Figure 2:**
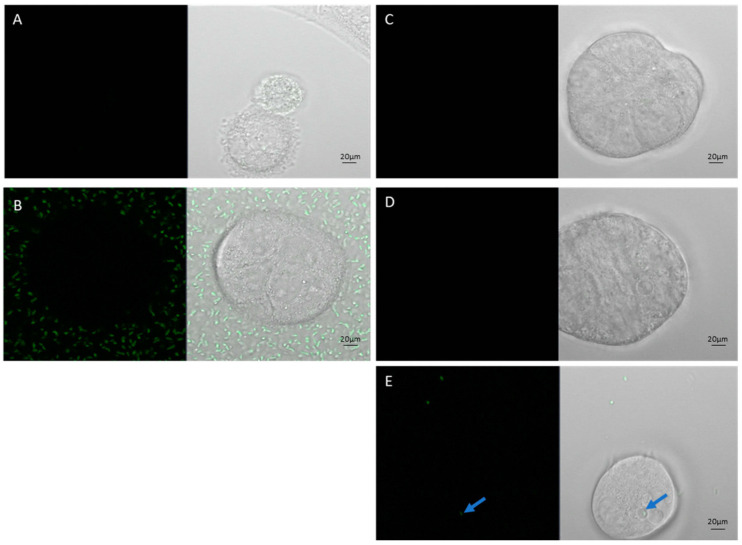
Caco-2 cells co-cultured with SL7207-GFP bacteria (green) in 2D and 3D Matrigel models: (**A**) Caco-2 2D no bacteria, (**B**) Caco-2 2D plus bacteria in media, (**C**) Caco-2 3D no bacteria, (**D**) Caco-2 Matrigel 3D plus bacteria in media, (**E**) Caco-2 Matrigel 3D plus bacteria mixed through gel. Blue arrow in (**E**) indicates a solo GFP bacteria that has survived and invaded in a Matrigel 3D CaCo-2 co-culture. Scale bars represent 20 µm.

However, once applied to a 3D co-culture system in a Matrigel ECM, poor survival or invasion was observed. With paired 2D and 3D experiments (Figure 2B,D), it was apparent that a component of the Matrigel ECM inhibited invasion by *Salmonella*. Matrigel contains gentamicin, a bactericidal aminoglycoside with good efficacy against *Salmonella*.

**Figure 3:**
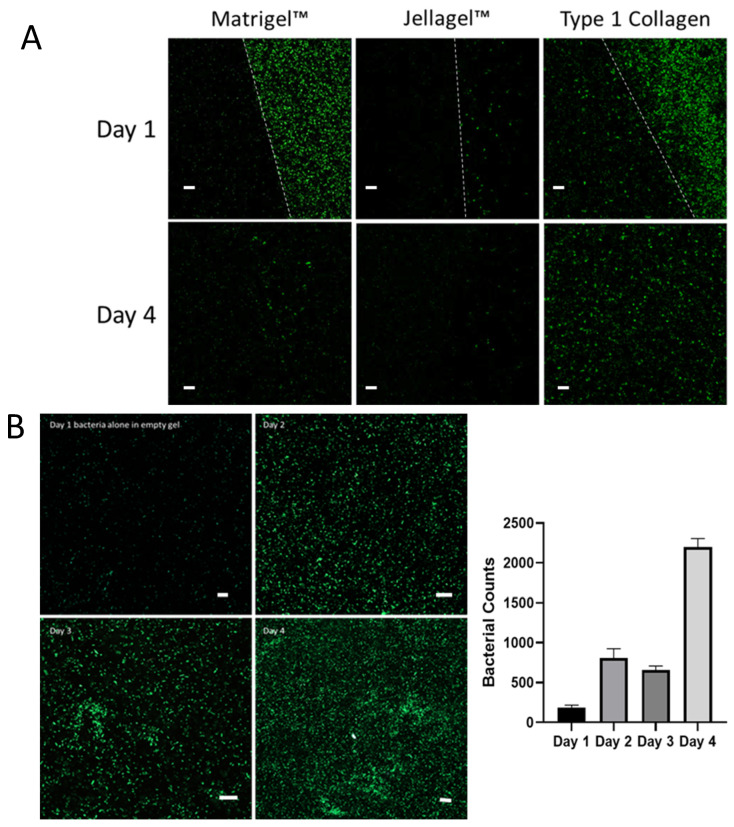
(**A**) SL7207-GFP bacterial survival and infiltration in Matrigel, Jellagel and type 1 collagen after 1 day and 4 days of incubation. After 1 day of incubation, SL7207-GFP bacteria (green) can be seen in the media of both the Matrigel and collagen, and some beginning to enter the collagen, but very few persisting with the Jellagel. The white-dotted lines represent the boundaries of the respective ECMs. At day 4, few bacteria remain in either the Matrigel or Jellagel wells but can be seen to have colonised and thrive within the collagen gel. (**B**) SL7207-GFP bacteria cultured in Type 1 Collagen showing increasing bacterial population over 4 days. The right-hand panel represents mean and standard deviation of the number of bacteria per 450 µm^2^ field at each time point (n = 4). Scale bars (white) represent 20 µm.

Therefore, we investigated other commercially avai lable collagen matrices for organoid cultures. Most mammalian-derived options contain antibiotics to prevent bacteria from the origin species being introduced to cell culture applications. This has the consequence of rendering them poor models for studying bacterial infection. Using an invertebrate-derived collagen, Jellagel, we also had limited success (Figure 3A). However, using rat tail-derived antibiotic-free type 1 collagen, we demonstrated survival of *Salmonella* SL7207-GFP and, indeed, the colonisation of the ECM (Figure 3A), with more than 5-times greater bacterial numbers compared to Matrigel on Day 1, increasing by Day 4 to a ratio of more than 8:1. The bacteria persisted and multiplied over four days in the absence of any cells in the type 1 collagen ECM, increasing 10-fold in number in this period, indicating that this ECM is permissive for both infiltration and subsequent bacterial growth (Figure 3B).

**Figure 4:**
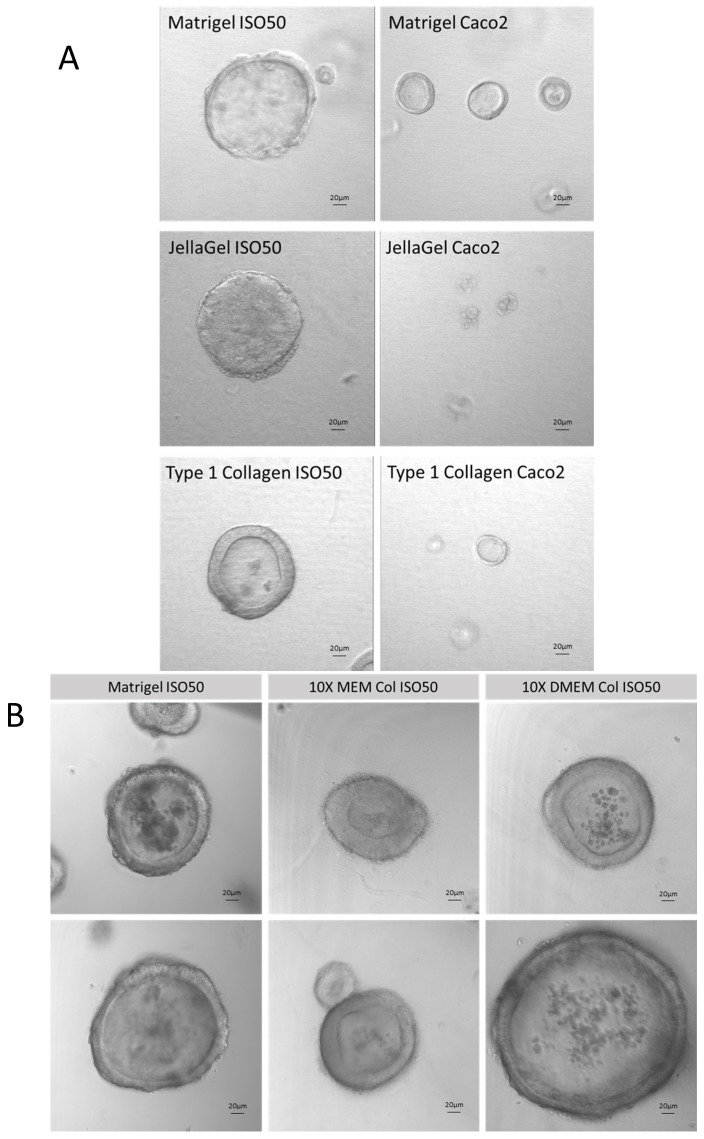
(**A**) Development of collagen type 1 Iso50 and Caco2 cell line organoids compared with Matrigel and Jellagel commercially available collagen matricies. (**B**) Optimisation of collagen type 1 organoid system in MEM and DMEM media showing good organoid structure compared to conventional Matrigel organoids.

In terms of the structural complexity of organoids, those grown using the type 1 collagen ECM could reproduce organoids prepared using Matrigel. Organoids prepared with ISO50 and Caco2 cells presented a distinct outer cell layer and an inner lumen with Matrigel, Jellagel and type 1 collagen. Initially, we observed organoids that were slightly smaller and the wall relatively thicker (Figure 4A) with type 1 collagen ECM. But further optimisation of the type 1 collagen organoid system with supplementation with either MEM or DMEM media resulted in organoid morphologies similar to those of organoids prepared with Matrigel (Figure 4B).

**Figure 5:**
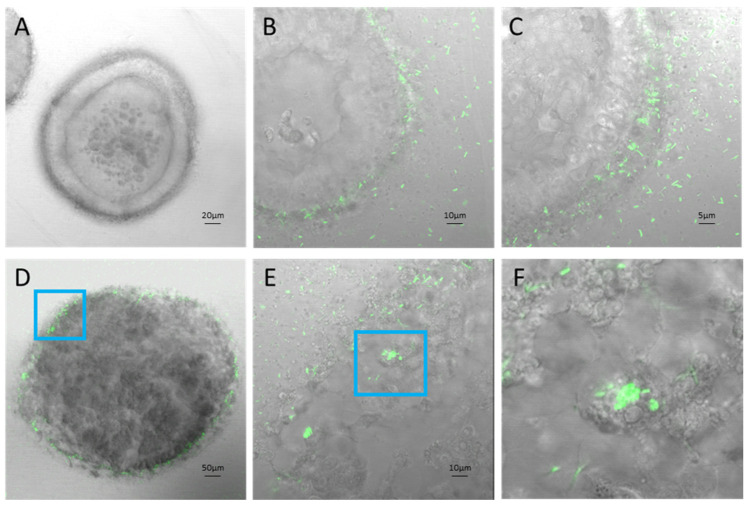
IS50 collagen organoids: (**A**) uninfected ISO50 in collagen gel, (**B**,**C**) SL7207 GFP infected ISO50 in collagen gel 1 h post-infection with visible GFP bacteria surrounding and invading organoid cells, (**D**) SL7207 GFP infected ISO50 in collagen gel 24 h post-infection, (**E**) Magnification of infected organoid cells, (**F**) 4× magnification of infected organoid cells (**E**).

Co-culture of Salmonella SL7207-GFP in ISO50 type 1 collagen organoids revealed chemoattraction to and invasion of the outer cell layers within an hour of bacterial application (Figure 5B,C). Further, 24 h after co-culture, SL7207 was seen to persist and replicate within the collagen organoid (Figure 5D–F). To illustrate that the *Salmonella* chemoattraction was driven by eukaryotic cellular factors, the co-culture was replicated with inert silicon beads in a collagen matrix. Bright light and fluorescence microscopy of SL7207-GFP in collagen gel culture with 0.1 mm silica sphere beads (https://www.mpbio.com/us/116914050-lysing-matrix-e-cf, (accessed on 7 March 2024)) showed no chemoattraction to the inert beads (Figure S2). The halo of aggregated SL7207 observed around colon cancer cell organoids was not observed with inert beads.

**Figure 6:**
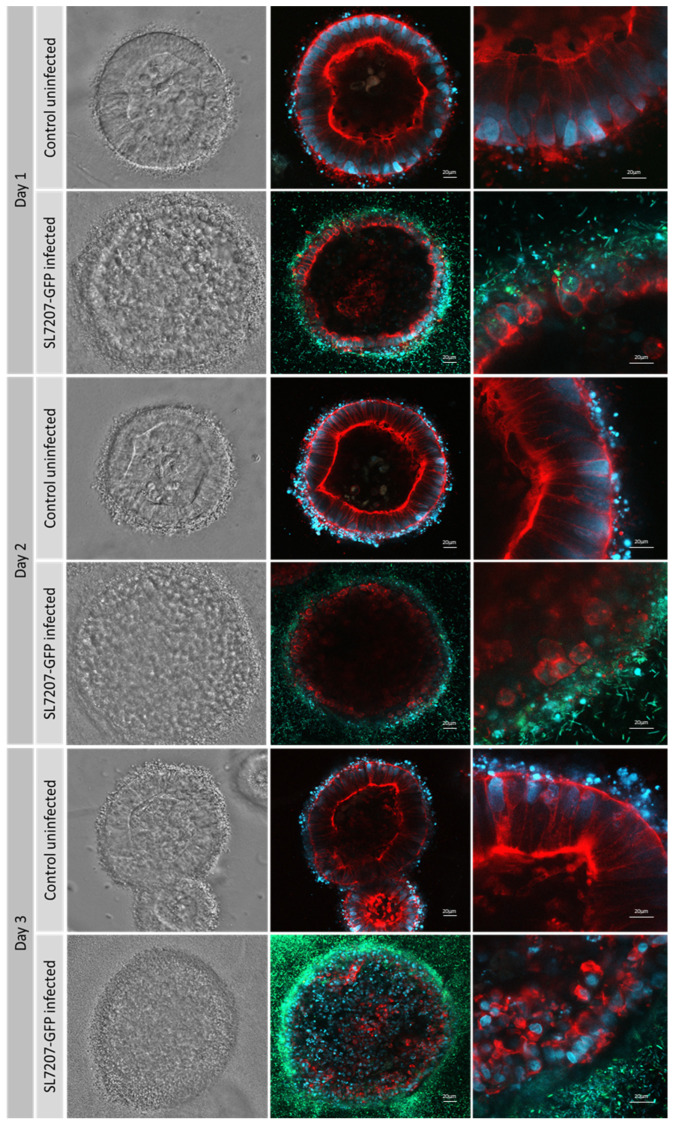
ISO50 collagen type 1 organoids with and without SL7207-GFP imaged under bright light and fluorescence confocal microscopy with ×20 and ×40 magnification. MOI of 2000:1 was used. Cells are stained with DAPI (blue), phalloidin (red), and GFP bacteria are green.

Confocal microscopy of ISO50 collagen type 1 organoids with and without SL7207-GFP imaged under bright light and fluorescence (MOI of 2000:1) revealed the invasion of the outer cell layers by day 1. This is particularly apparent when observed at 40× magnification (Figure 6, righthand panel). Cells were stained with DAPI (blue), phalloidin (red), and GFP bacteria (green). The quantification of the bacteria within the organoids indicated that between Day 1 and Day 3, the numbers effectively doubled. A halo of blue-staining material was observed around the uninfected organoids but was largely absent in the infected organoids.

**Figure 7:**
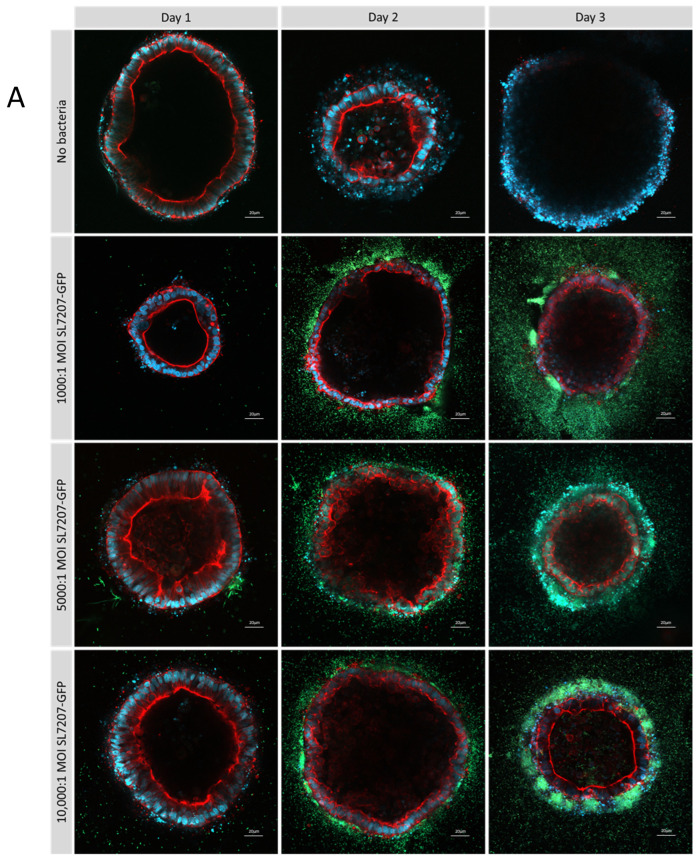

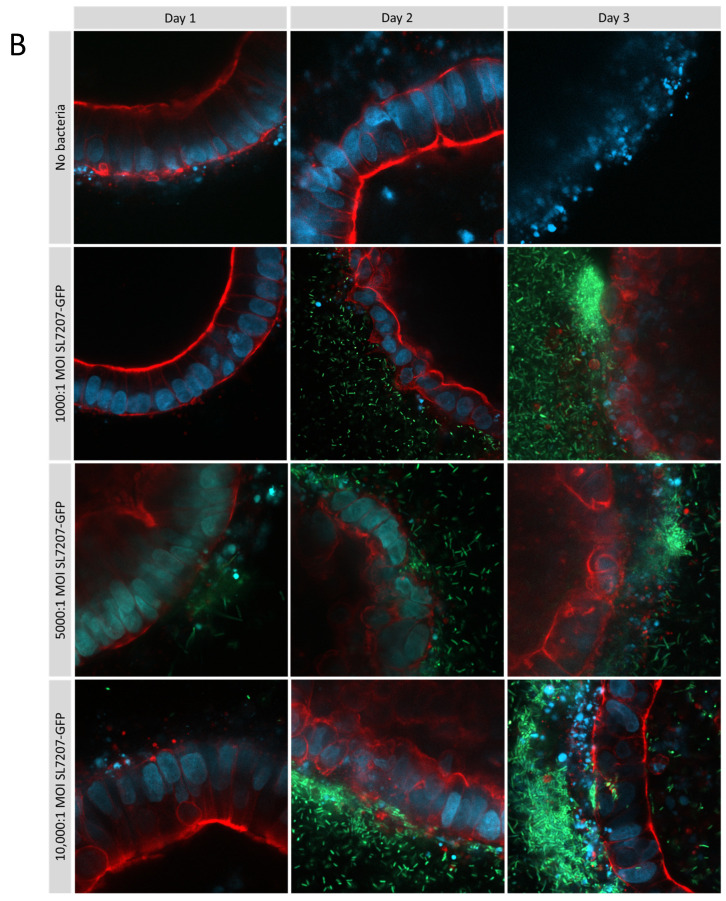
Optimisation of multiplicity of infection ratios in type 1 collagen ISO50 organoids treated with SL7207-GFP over 3 days. Cells are stained with DAPI (blue), phalloidin (red), and GFP bacteria are green and imaged by confocal microscopy at 20× magnification (**A**) and 40× (**B**) on a Zeiss LSM 880. After 3 days, a luminal population of SL7207-GFP can be observed particularly at MOI 10,000:1 (**B**).

Further optimisation of the multiplicity of infection ratios with type 1 collagen ISO50 organoids treated with SL7207-GFP over 3 days showed increased infection rates with increased bacterial dosage (Figure 7A), although these differences were not statistically significant. In a 2D co-culture model, high dosages, above an MOI of 4000:1, resulted in the rapid death of cancer cells, which prevents the observation of the effects of a high bacterial dosage. This demonstrates one of the advantages afforded by a 3D co-culture system. After 3 days, a luminal population of SL7207-GFP can be observed, particularly at an MOI of 10,000:1 (Figure 7B).

### 3.2. Patient-Derived Organoids

**Figure 8:**
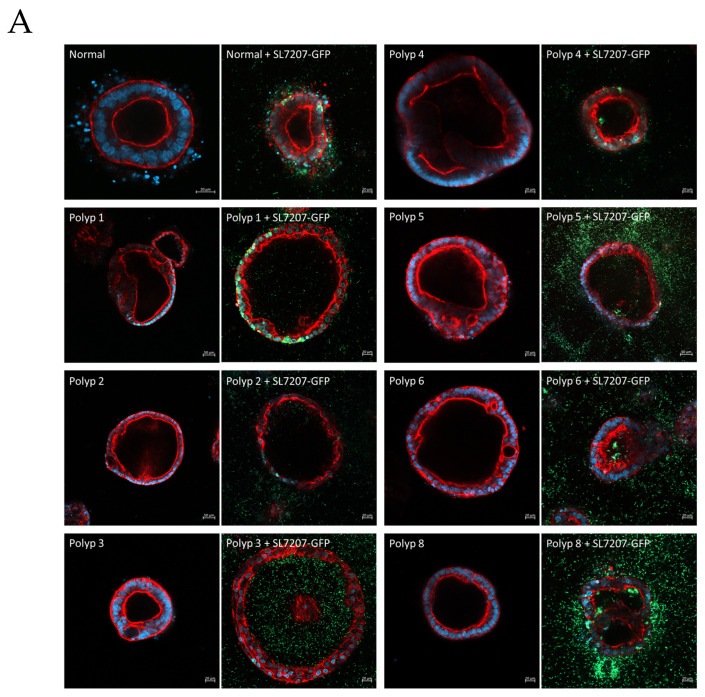

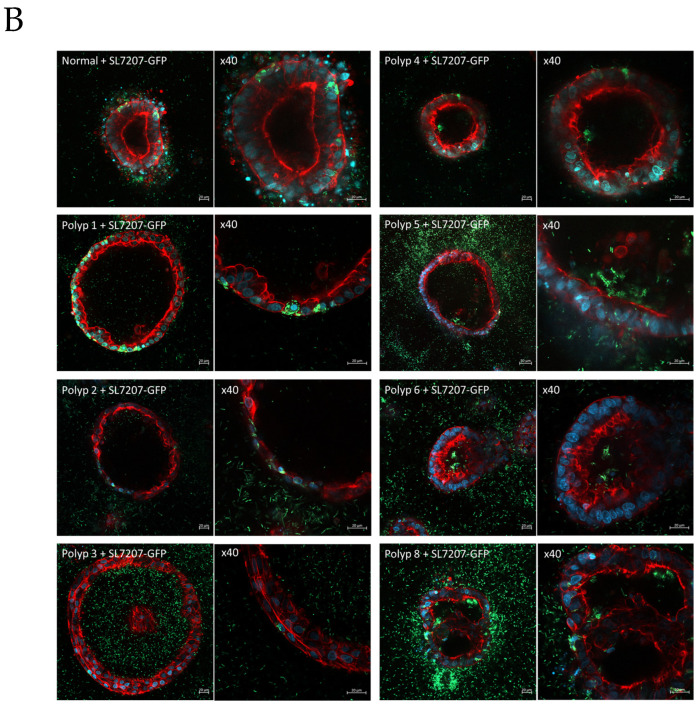
(**A**) Organoids derived from a single patient (SAPER0005) from normal tissue and multiple polyps. Organoids from each tissue sample/polyp are shown untreated and 24 h after treatment with SL7207-GFP. Cells are stained with DAPI (blue), phalloidin for actin (red), and GFP bacteria are green. (**B**) 20× and 40× magnification, intracellular organoid colonisation by the bacteria can be seen in both normal and polyp derived organoids, however intra-organoid (luminal) colonisation is only observed in polyp derived organoids.

Organoids derived from a single patient (SAPER0005) from normal tissue and multiple polyps were established in the type 1 collagen matrix (Figure 8A). Organoids from each tissue sample/polyp are shown untreated and 24 h after treatment with SL7207-GFP. With 20× and 40× magnification, intracellular organoid colonisation by the bacteria can be seen in both normal and polyp-derived organoids; however, intra-organoid (luminal) colonisation was only observed in polyp-derived organoids (Figure 8B).

The establishment and infection of a series of patient-derived organoids were demonstrated from another patient shown in Figure S3. SL7207-GFP infections of a patient (SAPER0006) were performed with organoids derived from (A) normal colon and (B), (C) and (D) polyps 1–3, respectively, with an MOI of 1000:1. Polyp organoids were observed to typically have thinner cell walls and a less conventional villus-like structure compared to normal tissue organoids. Green GFP-expressing bacteria can be seen in the cells of the organoid wall in the normal tissue organoid but have invaded through to the lumen in the polyp-derived organoids.

## 4. Discussion

Bacterial cancer therapy (BCT) offers a promising advancement in the treatment of many types of solid tumour. Indeed, recent progress in this area builds on much earlier discoveries that bacteria could cause the regression of tumours. For example, in the early 20th century, William Coley used live or heat-killed *Streptococcus pyogenes* and *Serratia marcescens* to treat patients with inoperable cancer, leading to a >10-year disease-free survival in 30% of patients [23]. More recently, attenuated mutants of several species of bacteria have been exploited for BCT, including mutants of cell-invasive *Salmonella eneterica* Serovar typhimurium that has been manipulated to produce and deliver therapeutic payloads to enhance their oncolytic properties [24,25,26,27]. Due to their attenuated phenotypes, these bacteria exhibit a high preference for tumour colonisation; for example, attenuated SL7207 *Salmonella*, which is deficient in the synthesis of aromatic amino acids, selectively colonises tumours that are a source of these nutrients, with a tumour-to-healthy tissue bacterial ratio exceeding 1000:1. Due to their motility and chemotaxis, the bacteria introduced to the circulatory system can efficiently target tumours and, once established, can proliferate within the immune-privileged tumour microenvironment (TME). This motility also allows the bacteria to migrate to and colonise hypoxic regions of the TME. In turn, the bacteria can utilise nutrients that would otherwise fuel tumour cell growth and division and offer intrinsic immunotherapy, leading to tumour regression. When combined with the ability of the bacteria to actively synthesise anti-cancer therapeutics, these properties can result in impressive outcomes in experimental tumour-bearing mouse models. However, to date, human clinical trials of BCT have been limited. One study on patients with metastatic melanoma used an attenuated *Salmonella* deficient in the synthesis of cell wall components and delivered by intravenous injection; some toxicity was observed at the highest dose (10^9^ cfu/m^2^) and, while demonstrating little or no toxicity at lower doses, any therapeutic effects were insignificant [28]. A second trial employed an attenuated *Salmonella*-expressing human interleukin-2. No toxic effects were observed, and a significant increase in circulating NK and NK-T cells indicated an immunologic response [29]. A promising study in dogs with metastatic osteosarcoma again demonstrated no toxicity but also improved survival due to orally administered *Salmonella* BCT [30]. A limitation to further clinical trials in humans is a knowledge gap in the precise understanding of the mode of action of BCT, including aspects concerning chemotaxis, tumour cell invasion and the responses of both tumour cells and the bacteria when the latter colonise the former. To address this, as has been done for other cancer therapeutics, it is vital to establish organoid models for BCT, as we did here. Organoids offer a reliable and predictive tool that can recapitulate the genomic, morphological and pathophysiological characteristics of a tumour. The use of organoids offers the potential for the animal-free optimisation of BCT, enabling the testing of bacterial mutants with improved characteristics with respect to chemotaxis, cell-invasive properties and reductions in any possible collateral effects.

The most widely used ECM for establishing organoids is Matrigel, but, as we demonstrate here, the inclusion of gentamicin in commercial Matrigel preparations inhibits bacterial growth and, thus, this ECM cannot be used to test BCT. Despite this, antibiotic-containing matrices are reported in recently published protocols for organoid/bacterial co-culture [7,31], although the bacteria have to be microinjected into the lumen to avoid being killed by the antibiotic in the ECM. In addition, Matrigel is a mouse tumour-derived ECM protein mixture with considerable batch-to-batch variation and is consequently not ideal for testing cancer drugs. Accordingly, we used an alternative type 1 collagen ECM to grow ISO50 organoids as models to investigate bacterial colonisation. As we demonstrate, supplementation with a defined growth medium such as DMEM permits the growth of organoids with similar morphologies to those grown in Matrigel. Co-culture of these organoids with *Salmonella* SL7207-GFP revealed no inhibition of the bacteria. The bacteria displayed a high level of chemotaxis towards the organoids, mimicking the tropism these bacteria exhibit in vivo. Subsequent steps in colonisation that we observed included the disappearance of DAPI-staining extracellular material, bacterial cell invasion and, ultimately, colonisation of the lumen. The size and morphology of the extracellular material are consistent, comprising nucleic acid-containing extracellular vesicles (EVs); the enhanced release of EVs was previously documented when a type 1 collagen ECM was used to grow colorectal cancer cell organoids [32]. The presence of the bacteria could either suppress the release of this material or, more likely, it is digested to fuel the growth of the bacteria; mammalian EVs have previously been shown to promote bacterial growth [33]. Clearly, this is one observation from this study that warrants further investigation as EVs are known to play a key role in cancer progression and metastasis [34], and any reduction in their number could be another facet of BCT not previously recognised.

A critical advancement for cancer therapy is the concept of personalised medicine, and patient-derived organoids offer a platform for testing therapeutic treatments designed for a specific patient tumour [35]. Here, we demonstrate the use of type 1 collagen for the establishment and infection of organoids derived from colorectal polyps excised from different patients. As with the ISO50 organoids, we observed chemotaxis, invasion and colonisation of the lumen in these patient-derived organoids. Of note is that the bacteria failed to colonise the lumens of organoids derived from healthy tissue, indicating abortive infection of these organoids. This is likely due to the normal regulated metabolism of the cells not providing sufficient aromatic amino acids to sustain bacterial growth, but, also, the aberrant morphologies of polyp-derived organoids may aid bacterial infection.

In conclusion, we demonstrate a key advance in using organoids grown using type 1 collagen as an ECM for in vitro studies of BCT. This advance will help with progress in optimising the bacterial chassis for tumour targeting, prior to any in vivo validation, providing critical information on mechanisms essential for regulatory approval of BCT and also with testing the efficacy of therapeutic payloads for personalized treatments that can be delivered by the bacteria. A specific limitation of these organoids prepared with type 1 collagen ECM is that, in the absence of an antibiotic in the ECM, they are susceptible to unwanted bacterial infection. General limitations, independent of the nature the ECM, include a lack of vasculature and an absence of infiltration by other cell types, notably immune cells. Advances in microfluidic devices and co-cultures with immune cells can potentially address these issues in the future [36].

## Figures and Tables

**Table 1:** Plasmids used in this study.

Plasmid	Attributes	Antibiotic Resistance Genotype	Reference
RSF1010	Low copy number	Streptomycin, sulfonamide	[17]
pLF300	Low copy number	Apramycin	This paper
pLF301	High copy number	Apramycin	This paper
pEG200	High copy number; GFP	Apramycin	This paper
pUC18-Apr	High copy number	Ampiciilin, apramycin	This paper
pUC18-Apr::RSF1010	High copy number	Ampicillin, apramycin, streptomycin, sulfonamide	This paper
pdagGFP	High copy number; GFP	Ampicillin	[18]
pIJ8600	High copy number	Apramycin	[19]

**Table 2:** Oligonucleotides used in this study.

Oligonucleotide	Sequence (5′-3′)
GFP-F	ATACTGCAGGATGGCTCTAGACTCGAAGG
GFP-R	GATCTGCAGCTCACTCATTAGGCACCCCAG
Apr (EcoRI)-F	ATCGAATTCAGCTCTCGGGTAACATCAAGG
Apr (EcoRI)-R	GAAGAATTCACATTATTTGCCGACTACCTTGG
Apr (BamHI)-F	ATCGGATCCAGCTCTCGGGTAACATCAAGG
Apr (BamHI)-R	GATGGATCCACATTATTTGCCGACTACCTTGG
02232021-F	ATGGCCCCCACCAGCACC
02232021-R	TTGGCCAAGGTGAACAGCAGC

## Data Availability

The original contributions presented in this study are included in the article/Supplementary Materials. Further inquiries can be directed to the corresponding authors.

## Supplementary Materials

The following supporting information can be downloaded at: https://www.mdpi.com/article/10.3390/cells14070524/s1, Figure S1: Derivation of a RSF1010-based plasmid for stable expression of GFP; Figure S2: Absence of bacterial chemoattraction to inert beads in a collagen ECM; Figure S3: Infection of 3 patient-derived polyp organoids; Table S1: ISO50 media.

## Abbreviations

The following abbreviations are used in this manuscript:

BCT	Bacterial cancer therapy
ECM	Extracellular matrix
Apr	Apramycin
PBS	Phosphate-buffered saline
PFA	Paraformaldehyde
IF	Immunofluorescence
GFP	Green fluorescent protein
EV	Extracellular vesicle
DAPI	4′,6-diamidino-2-phenylindole
