# Supplementary material for: Organoids with a Type 1 Collagen Scaffold to Model Bacterial Cancer Therapy
Source: Cells. 2025 Apr 1;14(7):524. doi: 10.3390/cells14070524 (PMC11989105; doi:10.3390/cells14070524)
Supplement: Supplementary file 1 [file cells-14-00524-s001.zip › cells-3511100-supplementary.pdf]

Organoids with a Type 1 Collagen Scaffold to Model Bacterial Cancer Therapy

Lydia Farrell, Cleo Bonnet, Alethea Tang, Severina Peneva, Non G. Williams, Sunil Dolwani, Lee Parry and Paul Dyson

Supplementary data

A B

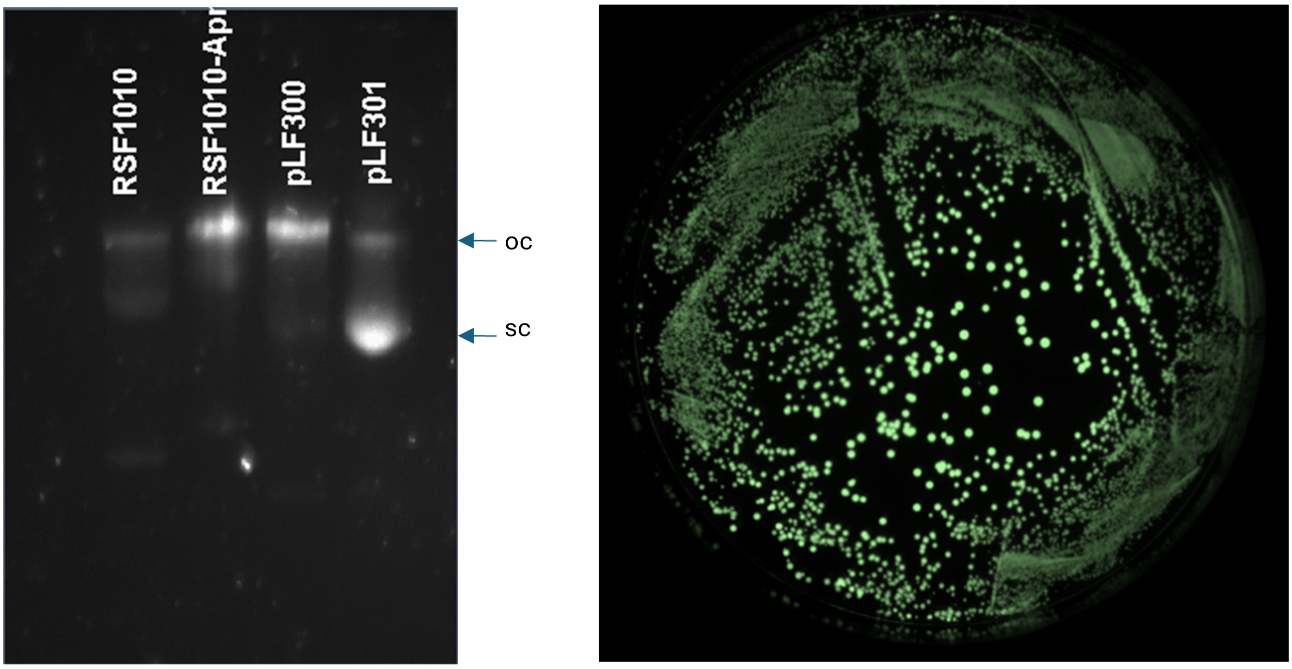

Figure S1. Derivation of RSF1010-based plasmids.

(A) As described, RSF1010 was manipulated to introduce an apramycin-resistance gene (RFS1010-Apr), adjacent non-essential sequences were deleted (pLF300), and subsequently a 4 bp deletion introduced into the *mobC* gene, resulting in plasmid pLF301. Agarose gel electrophoresis of plasmid isolated from equivalent culture volumes of bacteria revealed both increased copy-number and vastly improved yield of supercoiled (sc) DNA compared to nicked open-circle DNA (oc) of pLF301.

(B) A gfp gene under control of a tac promoter was subsequently included in plasmid pEG200. Colonies of SL7207 with pEG200 were imaged for fluorescence (BioRad Gel Doc) 0.3 sec.

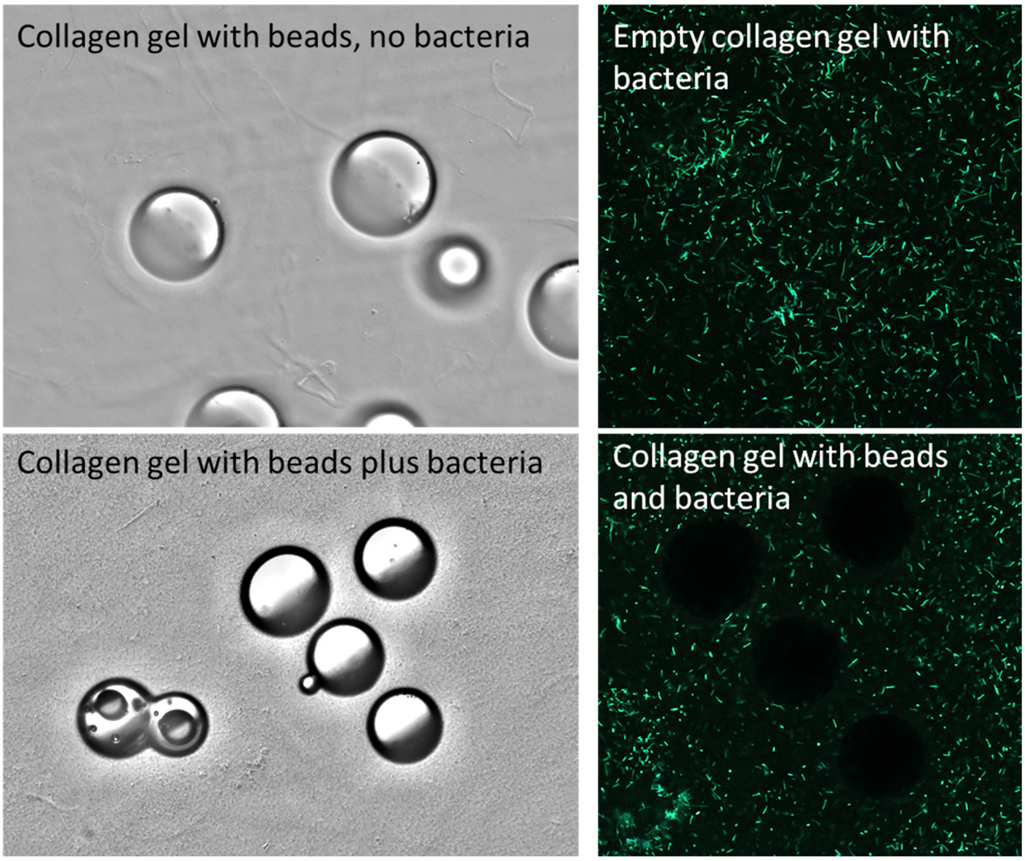

Figure S2. Bright light and fluorescence microscopy images of SL7207-GFP (green)in Collagen gel culture with silica beads demonstrating no chemoattraction to the inert beads, with the halo observed when cultured with cells being absent.

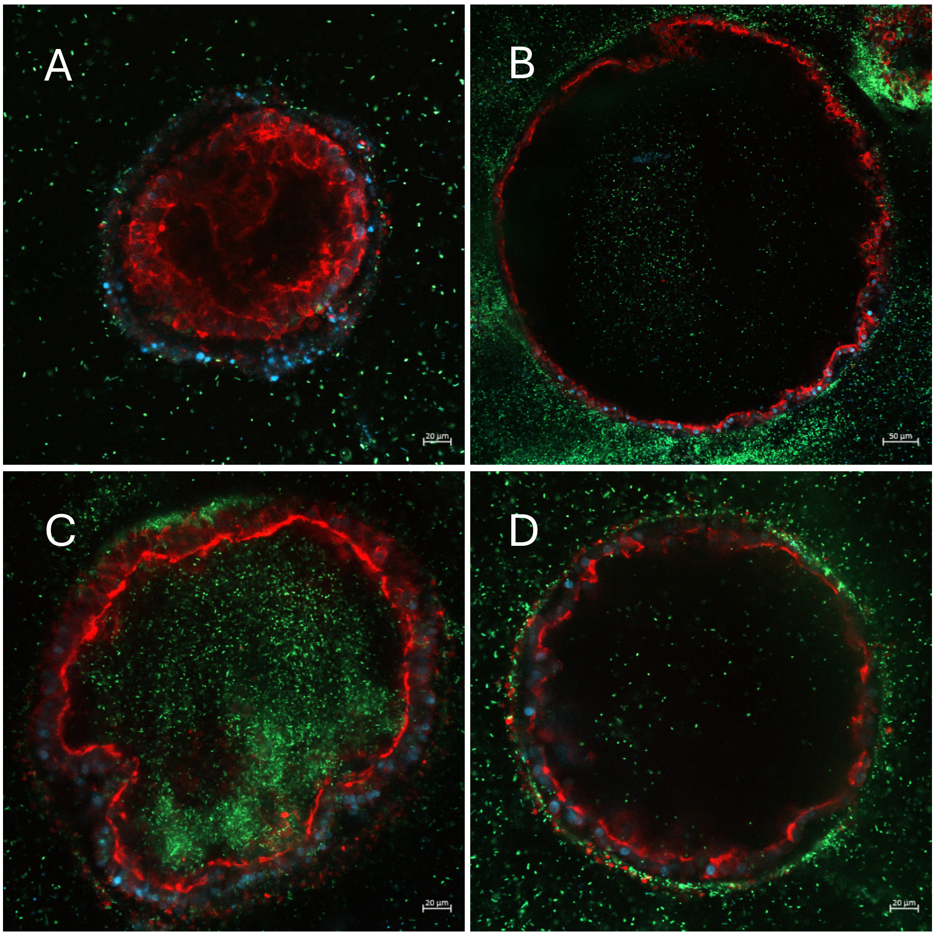

Figure S3. SL7207-GFP infections of patient (SAPER0006) derived organoids from A) Normal colon and B, C and D) polyps 1-3 respectively. MOI 1000:1. Cells were stained with DAPI (blue), Phalloidin (red) and the GFP bacteria are green. Polyp organoids have thinner cell walls and a less conventional villus-like structure compared to normal tissue organoid. Green GFP bacteria can be seen in the cells of the organoid wall in the normal tissue organoid (A), but not in the lumen as is evident in the polyp derived organoids.

Table S1

ISO50 Media:

Reagent	Concentration	Required Concentration	In 500ml (ml)	In 20ml (ml)
advanced DMEM/F12	-		500ml	20ml
Glutamax	100x	1x	5ml	200µl
Pen/Strep	10,000 U/ml	100U/ml	5ml	200µl
HEPES	1M	15mM	7.5ml	300µl
N2 supplement	100x	1x	5ml	200µl
B-27 supplement	50x	1x	10ml	400µl
n-acetyl cysteine	1M	1mM	200µl	8µl
Gentamycin	500x	1x	1ml	40µl
Fungizone	500x	1x	1ml	40µl
